# Pyrotinib Improves Survival in Previously Treated HER2-Positive Metastatic Breast Cancer

**DOI:** 10.1093/oncolo/oyac013

**Published:** 2022-03-28

**Authors:** Anne Jacobson

## Abstract

In the phase III PHOEBE trial, treatment with pyrotinib plus capecitabine significantly improved overall survival compared with lapatinib plus capecitabine in patients with previously treated HER2-positive metastatic breast cancer.

Approximately 15% to 20% of patients with breast cancer have tumors that harbor alterations in human epidermal growth factor receptor 2 (HER2). Although HER2-targeted therapies such as trastuzumab are the established standard for patients with HER2-positive breast cancer, resistance to HER2-targeted therapy inevitably develops.

Current options for patients with HER2-positive breast cancer that progresses on trastuzumab include lapatinib, a HER2-targeted tyrosine kinase inhibitor (TKI), in combination with capecitabine. Additional HER2-targeted therapies, such as trastuzumab emtansine (T-DM1), are emerging, although these agents are not approved for metastatic disease in many countries.

Pyrotinib is an oral, small-molecule, irreversible, pan-HER TKI that targets HER2, HER4, and HER1, also known as epidermal growth factor receptor. The phase III PHOEBE trial was designed to compare pyrotinib plus capecitabine with lapatinib plus capecitabine in patients with HER2-positive metastatic breast cancer previously treated with trastuzumab and taxanes-based chemotherapy.^1^

In the primary analysis of the PHOEBE trial, reported earlier this year, pyrotinib reached the primary endpoint of improved progression-free survival (PFS) relative to lapatinib.^1^ Binghe Xu, M.D., Ph.D., of the Chinese Academy of Medical Sciences, presented updated results from the PHOEBE trial, including an analysis of overall survival (OS).^2^

## Study Design

The phase III PHOEBE trial enrolled 267 patients from China with pathologically confirmed HER2-positive metastatic breast cancer. All patients had been previously treated with trastuzumab and taxanes, with a maximum of 2 lines of prior chemotherapy in the metastatic setting. Patients were randomly assigned to treatment with pyrotinib plus capecitabine (*n* = 134) or lapatinib plus capecitabine (*n* = 132). The primary endpoint was PFS.

Baseline characteristics were similar in both treatment groups (Table 1). Median patient age was approximately 50 years, and more than 70% of patients in each treatment arm had evidence of resistance to prior trastuzumab therapy.

**Table 1.** Baseline characteristics of patients with HER2-positive metastatic breast cancer

Characteristic	Pyrotinib plus capecitabine (*n* = 134)	Lapatinib plus capecitabine (*n* = 132)
Median age	50 years	49 years
ECOG performance status		
0	35%	33%
1	65%	67%
Hormone receptor status		
ER-positive and/or PR-positive	46%	44%
ER-negative and PR-negative	54%	56%
Metastatic sites at screening		
Visceral	77%	82%
Non-visceral	23%	18%
Previous trastuzumab therapy		
For advanced disease	59%	67%
As adjuvant or neoadjuvant therapy	56%	48%
Both	15%	15%
Median duration of prior trastuzumab for advanced disease	5.9 months	4.7 months
Resistance to prior trastuzumab		
Yes	72%	76%
No	28%	24%
Previous lines of chemotherapy for metastatic disease		
0	43%	35%
1	42%	49%
2	16%	16%

Abbreviations: ECOG, Eastern Cooperative Oncology Group; ER, estrogen receptor; PR, progesterone receptor.

## Key Findings

In the updated analysis, the median follow-up was 33.2 months in the pyrotinib plus capecitabine arm and 31.8 months in the lapatinib plus capecitabine arm. In total, 40.3% of patients in the pyrotinib arm and 52.3% in the lapatinib arm had died. This represents a 31% reduction in the risk of death among patients treated with pyrotinib relative to those treated with lapatinib (HR, 0.69; *p* =.02) (Table 2).

**Table 2.** Overall and progression-free survival in HER2-positive metastatic breast cancer

Endpoint	Pyrotinib plus capecitabine(n = 134)	Lapatinib plus capecitabine(n = 132)	HR(95% CI)	*p* value
Median OS	Not reached	26.9 months	0.69 (0.48-0.98)	.02
Median PFS	5.6 months	12.5 months	0.48 (0.37-0.63)	<.0001

Median OS was not reached in the pyrotinib arm, compared with 26.9 months in the lapatinib arm. The 24-month OS rates were 66.6% and 58.8% in the pyrotinib and lapatinib groups, respectively.

Pyrotinib was associated with a significant improvement in median PFS compared with lapatinib (5.6 months vs. 12.5 months). This represents a 52% reduction in the risk of disease progression with pyrotinib (HR, 0.48; *p* <.0001) (Table 2). The OS and PFS benefits of treatment with pyrotinib were consistent across most clinically relevant patient subgroups, including those with and without trastuzumab resistance. The analysis also favored pyrotinib regardless of the number of prior lines of chemotherapy.

The combination of pyrotinib plus capecitabine was associated with a manageable safety profile. The full safety analysis was reported previously.^1^

In 2020, based on initial findings from the PHOEBE trial, pyrotinib plus capecitabine was approved in China as second-line treatment for patients with HER2-positive metastatic breast cancer.^1^ The current updated results demonstrating improved OS support the use of pyrotinib as a standard of care in this treatment setting.

Xu and colleagues noted that pyrotinib may play a role in the second-line treatment of HER2-poisitve metastatic breast cancer in countries where access to novel HER2-directed therapies and antibody-drug conjugates such as pertuzumab or T-DM1 is limited.

## Notes

Please note that this content was created for the purpose of summarizing the content of the San Antonio Breast Cancer Symposium 2021 and has not been peer reviewed under the standard policies of *The Oncologist*.
